# Immunosuppressant-Induced Oxidative Stress and Iron: A Paradigm Shift from Systemic to Intrahepatic Abnormalities

**DOI:** 10.1155/2020/8675275

**Published:** 2020-03-31

**Authors:** Tasleem Akhtar, Gibran Ali, Nadeem Sheikh

**Affiliations:** ^1^Department of Pharmacology, University of Health Sciences, Lahore, Pakistan; ^2^Department of Physiology and Cell Biology, University of Health Sciences, Lahore, Pakistan; ^3^Cell and Molecular Biology Lab, Department of Zoology, University of the Punjab, Lahore, Pakistan

## Abstract

Immunosuppressants are used clinically to lower rejection rates in transplant patients. Unfortunately, the adverse side effects of these immunosuppressants can be severe, which is one of the rationales that life expectancy of individuals after transplant still significantly falls short of that of the general population. The current experimental setup was designed to analyze the tacrolimus-induced hepatic iron overload in Wistar rats. Four experimental groups were orally given 1 ml of aqueous suspension of tacrolimus (12 mg/kg) through oral gavage, and rats were sacrificed after 6, 12, 24, and 48 h of tacrolimus dose. Hepatic hepcidin expression was found to be significantly augmented along with the upregulation of *Tf* and *TfR1*, Ferritin-L, Ferritin-H, *TNF-α*, and *HO-1* gene expression at 6 and 12 h, and downregulation of *Fpn-1*, *Hjv*, and *Heph* at 6 h was detected. Significant downregulation of *IL-6*, *IFN-α*, *IFN-β*, and *IFN-γ* at all study time points was also observed. Serum iron level was decreased while serum hepcidin level was found to be significantly increased. Iron staining showed blue-stained hemosiderin granules within the hepatocytes, sinusoidal spaces, and portal areas at 12 and 24 h time points and remarkable fall of iron contents in the splenic red pulp. These results suggest that the use of tacrolimus leads to the onset of an intrahepatic acute-phase response-like reaction and causes iron overload in hepatic cells by altering the expression of key proteins involved in iron metabolism.

## 1. Introduction

Generally, transplantation is a lifesaving intervention for the patients suffering from organ failure at end stages and transplantation medication plot is one of the foremost complex and challenging area of a modern medical system [1]. The organ rejection is the major limitation factor in successful application of the technique, and that happens due to activated T-lymphocytes as a part of adaptive immune response. Patients after organ transplantation are forced to take lifelong immunosuppressive drugs to suppress the immunity and thus stabilize the transplant in the body of the patient [2]. Graft survival has improved significantly over the last few decades; nevertheless, late posttransplantation complications still present a growing challenge. All immunosuppressant used in transplant can be considered a high-risk medication. Tacrolimus is a pivotal immunosuppressive drug used clinically to lower the rate of immunological rejection after solid organ transplantation [3]. It is well known that its immunosuppressive possessions are dependent on calcineurin inhibition [4, 5]. Due to the inhibition of calcineurin, tacrolimus modifies several biochemical processes, which can lead to undesirable side effects [6, 7]. Anemia is common after transplantation, and immunosuppressants have long been involved in the pathogenesis of anemia after transplantation [8].

Iron status is a critical factor in patient-related outcomes in transplant medicine. Iron deficiency and/or iron overload have been supposed to be risk factors after organ transplantation [9]. The decrease in serum and the increase in hepatic iron uptake are the hallmark of acute-phase response (APR) [10]. According to an actual hypothesis, iron homeostasis is regulated by a large group of iron regulatory proteins including hepcidin (*Hepc*) which is a major acute-phase protein, ferroportin-1 (*Fpn-1*) which is a negative acute-phase protein, hemojuvelin (*Hjv*), ferritin, transferrin (*Tf*), and transferrin receptors (*TfR1*, *TfR2*) [11]. *Hepc*, a central regulator of iron homeostasis, is a liver-secreted peptide that binds to the sole iron exporter, *Fpn-1*, and causes its internalization and degradation [12]. Through this mechanism, *Hepc* decreases the circulating iron by blocking iron absorption via duodenal enterocytes and macrophage iron release. *Fpn-1* and a ferroxide that is hephaestin (*Heph*) play a collaborative role in iron transport from enterocytes. In the presence of *Fpn-1* and *Heph*, the newly released ferrous iron oxidized to its ferric form, which allows it to bind to *Tf* [10]. *Tf* and transferrin receptors are major proteins which take part in the transport and cellular uptake of iron. Plasma iron is majorly bound with *Tf*, which is an abundant iron-binding protein. The majority of the cells fulfill their iron need by taking iron-bounded *Tf* from the plasma and extracellular fluids via a transferrin receptor 1- (*TfR1*-) mediated process [13]. Both monoferric and diferric *Tf* move towards endosomes, where low pH detaches iron from the receptor-ligand complex. Then, iron-free *Tf* is transferred back to the cell membrane which is further released into the plasma at neutral pH, and *TfR1* becomes ready to enter the next cycle of iron uptake [14, 15]. Because *TfR1* is ubiquitously expressed, *Tf*-mediated iron uptake is considered to occur in the majority of cell types [16]. Ferritin-H and Ferritin-L subunits are accountable for iron storage within the hepatic cells [17]. The two subunits are regulated differentially and independently of each other at the transcriptional and posttranslational levels [18]. Ferritin level remains the primary mean of clinically assessing iron overload; however, it is to be considered that the inflammatory response may be accompanied by elevated ferritin levels [19]. Heme oxygenase (*HO*) is a ubiquitously expressed enzyme responsible for the degradation of heme. The expression of intracellularly located *HO-1* is induced by cellular stress, such as elevated levels of prooxidants by inflammatory stimuli. *HO-*1 is induced in vascular endothelial cells by the cytokine, tumor necrosis factor-*α* (*TNF-α*), and plays a significant part in mediating the proinflammatory effect of *TNF-α* [20, 21].

To date, there is no published data reporting dysregulation of iron metabolism by use of tacrolimus in an animal model. This study was aimed at investigating the induction of changes in the expression of the key genes involved in iron metabolism generated by hepatotoxic potential of tacrolimus. Our results clarify hematologic effects of tacrolimus, indicating this immunosuppressant as a potential cause of impaired *Hepc* production and iron overload in hepatic cells after transplantation.

## 2. Methods

### 2.1. Animals and Treatment

45 adult male Wistar rats of twelve to fourteen weeks of age, weighing 250 ± 25 g, were used in this project. Prior to experimentation, the rats were housed 5 per cage and kept under controlled environmental conditions. Rats were given free access to standard rat laboratory diet and tap water. This study was performed in accordance with the guidelines for the care and experimentation protocol of laboratory animals approved by Local Ethical and Review Committee of the Department of Zoology, University of the Punjab, Lahore. Nine animals were used as the control and thirty-six as experimental (nine for each time point). Four experimental groups were orally given 1 ml of aqueous suspension of tacrolimus powder (12 mg/kg) through oral gavage. Normal drinking water was given to control rats, and animals were euthanized by an overdose of ether after 6, 12, 24, and 48 h of tacrolimus suspension administration. All the animals were anesthetized by using an equal ratio of ketamine plus pyrogen-free water intraperitoneally. Blood was collected through direct cardiac puncture for serum separation under anesthesia conditions. After euthanizing by an overdose of ether, liver lobes were removed and divided into two parts: the first part was snap frozen at -80°C for genomic analysis and the second part was fixed in 10% formalin for histochemical analysis. The spleen was also removed and fixed in 10% formalin for histochemical analysis.

### 2.2. RNA Isolation

Snap-frozen liver samples were used for total RNA extraction by using a TRIzol method. RNA samples were quantified via a spectrophotometer (NanoDrop, ND-1000). Total RNA concentration was calculated by measuring the absorbance at A260. The ratio of A260/A280 is used to evaluate the purity of RNA. A ratio of 1.8-2.0 was accepted as pure.

### 2.3. Quantitative Real-Time PCR

cDNA was generated via 1.5 *μ*g of total RNA by using the Fermentas reverse transcription kit (cat#K1632) according to the manufacturer's instructions. Expressions of different genes (*Hepc*, *Fpn-1*, *Hjv*, *Heph*, *Tf*, *Tfr1*, Ferritin-L, Ferritin-H, *HO-1*, *TNF-α*, *IL-6*, *IFN-α*, *IFN-β*, and *IFN-γ*) were examined by using Maxima SYBR Green qPCR Master Mix. *β*-Actin was used for normalization (housekeeping gene). Gene-specific primer sequences are listed in Table 1. The assay of all the samples was done in triplicate. The curves of amplification were analyzed to measure the Ct value.

### 2.4. Measurement of Serum Iron Levels

A colorimetric method is used in which ferric iron (Fe^+3^) is released from its carrier protein, transferrin, in an acid medium (pH 4.0) and simultaneously reduced to the ferrous form (Fe^+3^) by ascorbic acid. The ferrous iron is then bound to chromogen, a sensitive iron indicator, to form a blue-colored chromophore which absorbs maximally at 595 nm.

### 2.5. ELISA

ELISA, which is a solid-phase enzyme-amplified sensitivity immunoassay, was used for quantitative detection of serum *Hepc* level using the commercially available specific enzyme-linked immunosorbent assay kit (BioSource International). Both intra-assay and interassay variations were less than 15% and a sensitivity of 1.0 ng/ml for *Hepc* assay. All the serum samples were analyzed in triplicate.

### 2.6. Histochemical Analysis

Liver and spleen tissues were fixed in 10% formalin, dehydrated in different grades of ethyl alcohol in ascending order of their strengths (40%-100%), cleared in xylene, and embedded in a paraffin wax followed by sectioning. Sections (5 *μ*m thick) were stained with Prussian blue iron stain from Sigma-Aldrich. For staining, a standard protocol was followed with minor modifications as described by [22]. Briefly, slides having sections were deparaffinated and tissues were rehydrated with deionized water for about five minutes. Slides were placed in working iron stain solution for 15 minutes followed by three washes in deionized water. For counterstaining, slides were subsequently stained in nuclear fast red working solution for 10 minutes and rinsed in deionized water followed by dehydration through alcohol and tissue clearance by xylene.

### 2.7. Statistical Analysis

Data were evaluated using Prism GraphPad 5 software (San Diego, CA). One-way analysis of variance (ANOVA) and Tukey's *post hoc* test were performed to identify any significant differences between the groups. *P* values less than 0.05 were considered significant.

## 3. Results

### 3.1. Changes in mRNA Expression of Iron Regulatory Proteins

**Figure 1:**
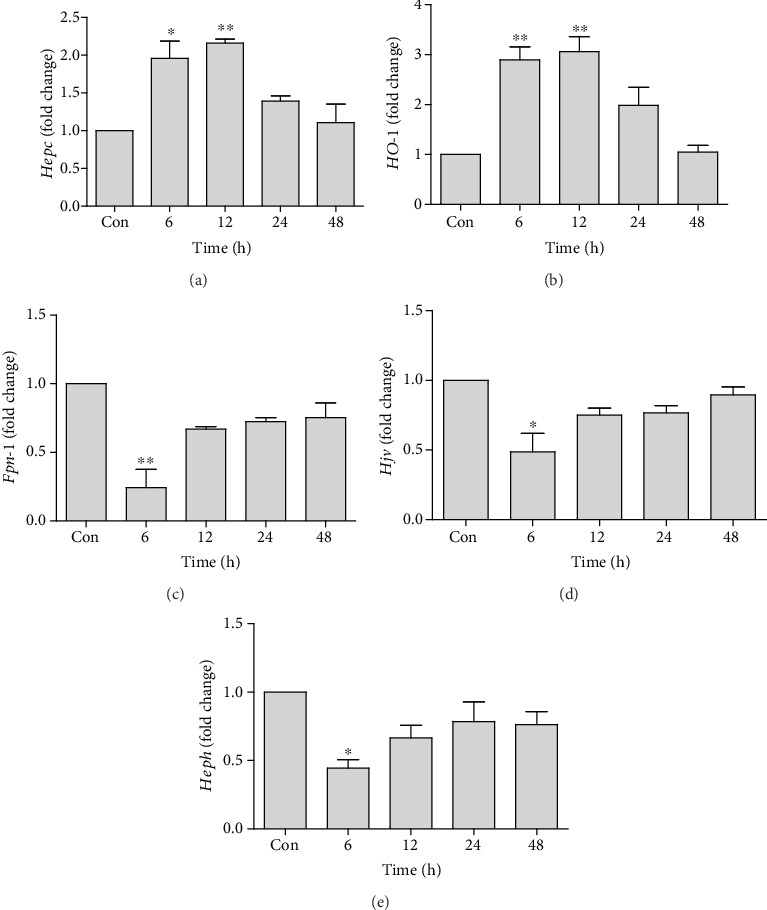
Quantification of mRNA expression of *Hepc* (a), *HO-1* (b), *Fpn-1* (c), *Hjv* (d), and *Heph* (e) in tacrolimus-induced toxicity at different time points in relation to the control. qRT-PCR data were shown as fold change in the expression of mRNA, normalized with *β*-actin (housekeeping gene). Values represent the mean ± S.E.M., analyzed by one-way ANOVA. ^∗^*P* ≤ 0.05 and ^∗∗^*P* ≤ 0.01 when compared to the control.

The expression of different iron regulatory genes was analyzed at different time points to assess the disturbances in iron metabolism. An initial increase in *Hepc* expression was observed at 6 h (1.95 ± 0.2-fold) and 12 h time points (2.1 ± 0.05-fold) after administration of tacrolimus. After 12 h, *Hepc* expression starts to decline towards baseline level (Figure 1(a)). A significant increase was noted in *HO-1* expression at 6 h (2.89 ± 0.25-fold) which reached a peak at 12 h (3.06 ± 0.30-fold), and after 12 h, *HO-1* expression starts to decline towards baseline level, while a significant downregulation of *HO-1* expression was observed at 48 h time point (1.047 ± 0.23-fold) as compared to 12 h time point (3.06 ± 0.30-fold) upregulation when analyzed by Tukey's *post hoc* test (Figure 1(b)). *Fpn-1* gene expression was found to be significantly downregulated at 6 h (0.24 ± 0.18) as compared to control animals. Intergroup comparison showed a significant upregulation in the expression of *Fpn-1* at 24 h (0.72 ± 0.04-fold) and 48 h (0.75 ± 0.15-fold) as compared to 6 h (0.24 ± 0.18) downregulation (Figure 1(c)). Similarly, *Hjv* and *Heph* gene expressions (0.48 ± 0.19 and 0.44 ± 0.08, respectively) were also downregulated at 6 h time point as compared to control animals when analyzed by one-way ANOVA, while intergroup comparison showed a significant upregulation of *Hjv* at 48 h (0.89 ± 0.07-fold) time point as compared to early downregulation in the expression at 6 h (0.48 ± 0.19-fold) time point (Figures 1(d) and 1(e)).

### 3.2. Changes in mRNA Expressions of Iron Import Proteins

**Figure 2:**
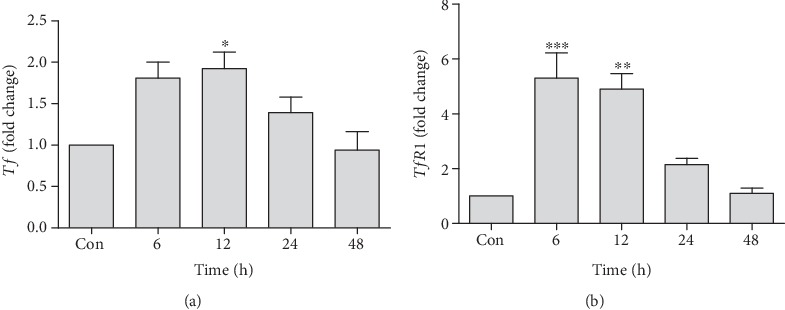
Quantification of mRNA expression of *Tf* (a) and *TfR1* (b) in tacrolimus-induced toxicity at different time points in relation to the control. qRT-PCR data were shown as fold change in the expression of mRNA, normalized with *β*-actin (housekeeping gene). Values represent the mean ± S.E.M., analyzed by one-way ANOVA. ^∗^*P* ≤ 0.05, ^∗∗^*P* ≤ 0.01, and ^∗∗∗^*P* ≤ 0.001 when compared to the control.

Expression of *Tf* and *TfR1*, which are involved in the transport and cellular uptake of iron, was also found to be upregulated. *Tf* expression was significantly upregulated reaching to the maximum at 12 h (1.92 ± 0.19-fold). Early upregulation at 6 h (1.81 ± 0.19-fold) was also observed, but this difference remained statistically nonsignificant. After 12 h, *Tf* expression starts to decline towards baseline level. This decline was statistically significant at 48 h (0.93 ± 0.38-fold) as compared to 12 h (1.92 ± 0.19-fold) time point upregulation (Figure 2(a)). A rapid and significant upregulation in the expression of *TfR1* was noted at 6 h (5.30 ± 0.9-fold) and 12 h (4.89 ± 0.56-fold) time points as compared to the control, while intergroup comparison showed a significant downregulation in the expression of *TfR1* at 24 h (2.14 ± 0.32-fold) and 48 h (1.099 ± 0.32-fold) time points as compared to early hour upregulation (Figure 2(b)).

### 3.3. Changes in mRNA Expression of Iron Storage Proteins

**Figure 3:**
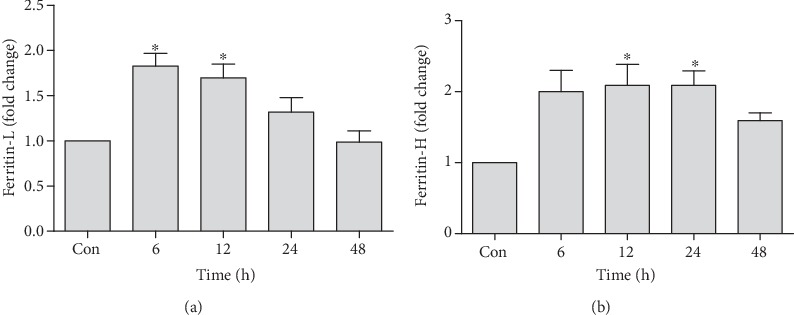
Quantification of mRNA expression of Ferritin-L (a) and Ferritin-H (b) in tacrolimus-induced toxicity at different time points in relation to the control. qRT-PCR data were shown as fold change in the expression of mRNA, normalized with *β*-actin (housekeeping gene). Values represent the mean ± S.E.M., analyzed by one-way ANOVA. ^∗^*P* ≤ 0.05 when compared to the control.

Ferritin-L expression found to be significantly upregulated at 6 h (1.82 ± 0.14-fold) and 12 h (1.69 ± 0.15-fold) time points in response to tacrolimus dose, and after 12 h, this upregulation started to decline towards baseline level. This decline was statistically significant at 48 h (0.98 ± 0.21-fold) as compared to early hour upregulation (Figure 3(a)). Ferritin-H expression was significantly upregulated at 12 h (2.08 ± 0.29-fold) and 24 h (2.08 ± 0.20-fold) time points as compared to the control. However, Ferritin-H showed a similar trend of upregulation at 6 h time point (1.99 ± 0.30-fold), though it was found statistically nonsignificant after the analysis with Tukey's *post hoc* test (Figure 3(b)).

### 3.4. Changes in mRNA Expression of Cytokines and Other Inflammatory Proteins

**Figure 4:**
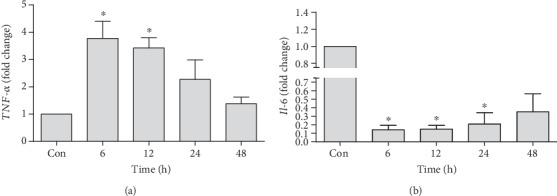
Quantification of mRNA expression of *TNF-α* (a) and *IL-6* (b) in tacrolimus-induced toxicity at different time points in relation to the control. qRT-PCR data were shown as fold change in the expression of mRNA, normalized with *β*-actin (housekeeping gene). Values represent the mean ± S.E.M., analyzed by one-way ANOVA. ^∗^*P* ≤ 0.05 and ^∗∗^*P* ≤ 0.01 when compared to the control.

**Figure 5:**
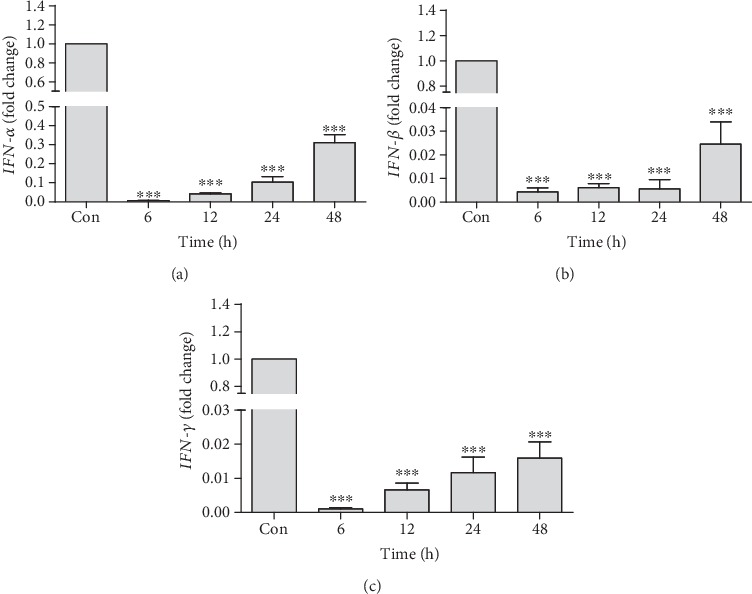
Quantification of mRNA expression of *IFN-α* (a), *IFN-β* (b), and *IFN-γ* (c) in tacrolimus-induced toxicity at different time points in relation to the control. qRT-PCR data were shown as fold change in the expression of mRNA, normalized with *β*-actin (housekeeping gene). Values represent the mean ± S.E.M., analyzed by one-way ANOVA. ^∗∗∗^*P* ≤ 0.001 when compared to the control.

*TNF-α* was also analyzed to assess the acute hepatotoxic potential of tacrolimus. A rapid upregulation of *TNF-α* was observed at 6 h (3.77 ± 0.6-fold) and 12 h (3.2 ± 0.30-fold) time points as compared to the control. Intergroup comparison showed a significant downregulation in the expression of *TNF-α* at 48 h (1.38 ± 0.42-fold) as compared to 6 h (3.77 ± 0.6-fold) upregulation (Figure 4(a)). At the same time, however, a rapid and significant decrease in the expression of *IL-6* expression was observed throughout the study (Figure 4(b)). Similarly, a quick and highly significant downregulation of *IFN-α* (Figure 5(a)), *IFN-β* (Figure 5(b)), and *IFN-γ* (Figure 5(c)) gene expression was observed throughout the planed experimental time points.

### 3.5. Changes in the Serum Iron Levels

**Figure 6:**
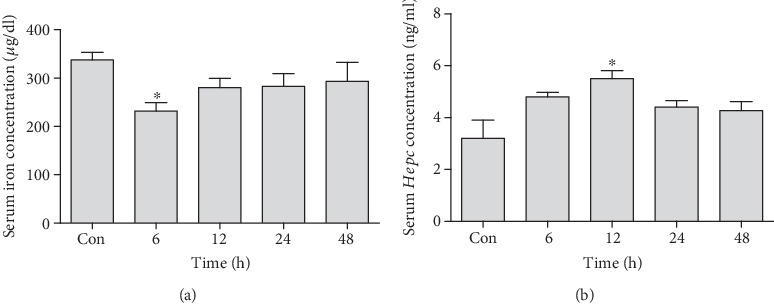
Time-dependent alterations in serum iron concentration (a) and serum *Hepc* concentration (b) in the experimental animals after the dose of tacrolimus in relation to control animals. Values represent the means of 4 replicates ± S.E.M., analyzed by one-way ANOVA. ^∗^*P* ≤ 0.05 when compared to the control.

Serum iron level was found to be significantly decreased at 6 h (231.7 ± 30.02) time point (*P* < 0.05) as compared to the control group (337.8 ± 31.74) when analyzed by Tukey's *post hoc* test, while there was no statistically significant difference in the levels of serum iron between different time points (Figure 6(a)).

### 3.6. Changes in Serum Hepcidin Levels

Serum *Hepc* concentration was measured via quantitative sandwich ELISA. A significant elevation in serum *Hepc* concentration was observed at 12 h (5.50 ± 0.59) time point (*P* < 0.05) as compared to the control group (3.20 ± 0.98). Concentration of *Hepc* was also elevated at 6 h time point, but this difference remained statistically nonsignificant when analyzed by Tukey's *post hoc* test, while there was no statistically significant change in the levels of serum *Hepc* between different time points (Figure 6(b)).

### 3.7. Prussian Blue Iron Staining of Liver and Spleen Tissues

**Figure 7:**
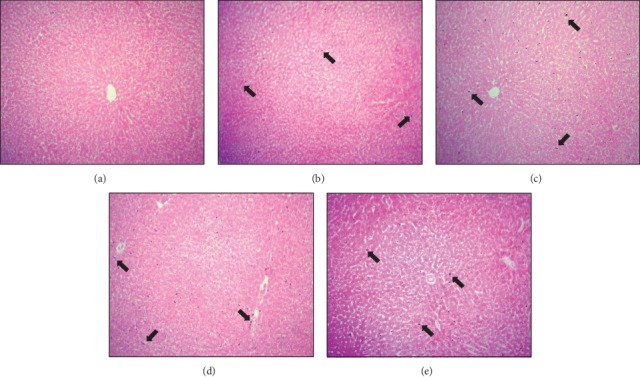
Microphotographs of Prussian blue iron-stained liver sections of the control (a) and tacrolimus-induced APR at 6 h (b), 12 h (c), 24 h (d), and 48 h (e) time points. Note iron deposition is dark blue in color (arrow), indicating the presence of iron in hepatocytes, sinusoidal spaces, and portal areas after the dose of tacrolimus in experimental groups as compared to the control.

**Figure 8:**
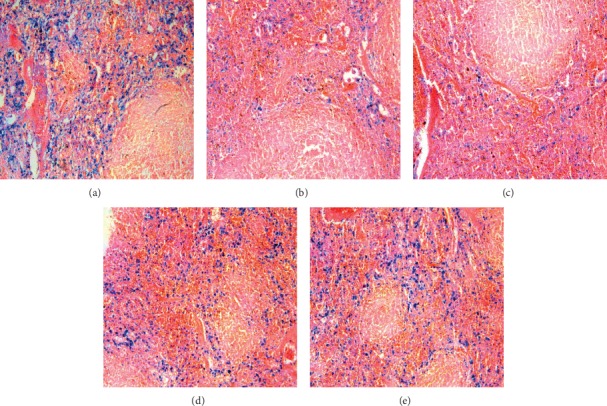
Microphotographs of Prussian blue iron-stained splenic sections of the control (a) and tacrolimus-induced APR at 6 h (b), 12 h (c), 24 h (d), and 48 h (e) time points. Iron contents are dark blue in color. Splenic sections after the use of tacrolimus revealed remarkable fall of iron contents in the red pulp at all experimental time points in comparison to control tissues.

No significant hemosiderin granules were found in hepatocytes, Kupffer cells, and sinusoidal spaces of hepatic sections of control rats (Figure 7(a)). After 12 and 24 h time points, blue-stained hemosiderin granules were observed within the hepatocytes, sinusoidal spaces, and portal areas. These clearly noticeable bluish granules were directed consideration towards the storage of iron in the liver (Figures 7(c) and 7(d)). A mild level of iron deposition was also observed at 48 h time point (Figure 7(e)). In contrast, splenic iron showed a tendency to decrease after 6, 12, and 24 h time points as compared to control sections (Figures 8(b)–8(d)). Iron staining revealed remarkable fall of iron contents in the splenic red pulp, which is rich in mononuclear cells, whereas the white pulp showed hardly any iron accumulation.

## 4. Discussion

This study was started to analyze the effect of tacrolimus on iron homeostasis. Tacrolimus works by binding to an immunophilin protein and inhibiting phosphatase activity of calcineurin in T-lymphocytes and lowers the risk of organ rejection [23]. Calcineurin inhibitors (CNIs) are a group of drugs which are given to pre- and posttransplantation to decrease the risk of rejection. With the use of CNIs as immunosuppressive agents, the risk of rejection has been reduced, but still, adverse effects of lifelong immunosuppression are a major concern [24].

Oral administration of aqueous suspension of tacrolimus in rats induced significant variations in the gene expression of key proteins considered to be involved in iron regulation. In the present work, *Hepc* expression was found to be significantly upregulated at 6 and 12 h time points. As *Hepc* is an acute-phase reactant, its expression is raised in conditions of liver damage and iron overload. It is also upregulated in conditions considered to overwhelm Kupffer cell engulfment capacity. Increased hepatic and nonhepatic *Hep*c expression was also described in turpentine oil-induced APR in rats [15], and also, upregulation of *Hepc* expression was indicated in response to hepatic damage *in vivo* and *in vitro* [12]. Pigeon and his colleagues, in 2001, first time described the relationship between *Hepc* and iron metabolism [25]. A previous literature also linked the upregulation of *Hepc* expression and iron-deficiency anemia [26]. *Hepc* is therefore considered to play a role as a negative regulator of iron absorption, recycling, and release from stores [14]. *Fpn-1* gene expression was downregulated in our study as demonstrated by the reduction of the specific RNA preceding upregulation of *Hepc* gene expression. This downregulation was parallel to the significant decline in serum iron levels. The signal for downregulation of *Fpn-1* was possibly linked with *TNF-α* upregulation. Previously, *Fpn-1* was found to be downregulated when bound with *Hepc*, as *Hepc* leads to internalization and degradation of this iron exporter protein [27].

Downregulation of *Hjv* and *Heph* expression in this study confirms previous studies [12, 15, 28] in different rat models of extrahepatic and intrahepatic APR that *Hepc* and *Hjv*-*Heph* gene expression together with *Fpn-1* changes simultaneously and in an opposite direction. The same results occurred in rats after oral administration of tacrolimus; an early upregulation of *Hepc* gene expression associated with time-dependent downregulation of *Fpn-1*, *Hjv*, and *Heph* gene expression was observed. Early upregulation of *Tf* gene expression was observed in this study. Expression of *TfR1* together with *TfR2*, accountable for iron uptake from diferric *Tf* through receptor-mediated endocytosis [15], was upregulated. Plasma iron deficiency was reported to have an effect on the stimulation of transcriptional activity of the hepatic *Tf* gene. A previous study conducted on patients with hepatic siderosis indicated that iron upholds stimulate *Tf* gene activity even when cellular iron contents are significantly increased [29]. A decline in the level of serum iron contents and successive raise in hepatocellular iron level is also a major characteristic of APR [11]. In our study, upregulation of both Ferritin-H and Ferritin-L chains was observed. This upregulation further confirms the hepatic iron storage. Hepatic ferritin gene expression is stimulated by iron overload which was previously observed in many *in vitro* and animal model studies [30].

Oxidative stress, cytokines, xenobiotics, and other mediators produced during inflammatory processes are mainly responsible for *HO-*1 induction [31]. In the current study, upregulation of *HO-*1 gene expression could be linked with the tacrolimus-induced “inflammatory” condition in the liver. High levels of *HO-*1 are repeatedly noticed in a variety of pathological states and generally in conditions containing oxidative stress at cellular level [32]. A rapid upregulation of intrahepatic *TNF-α*, one of the major acute-phase cytokines at early time points, was also observed in the current study confirming the inflammatory condition [33].

A quick and highly significant downregulation of *IL-6*, *IFN-α*, *IFN-β*, and *IFN-γ* was observed throughout the planned experimental time period. This downregulation may be one of the main immunosuppressive functions of tacrolimus acting directly in the transplanted organ. On the other hand, the immunosuppressant tacrolimus is a very powerful suppressor of T-lymphocyte activation, and at the same time, activated T-cells and NK cells are considered to be major contributors of *IL-6* and interferons [34]. Our data of histochemical analysis revealed that in the conditions of acute iron overload, iron was rapidly deposited in hepatocytes. During toxic conditions, a diversion of iron transfer occurs; i.e., iron accumulates not only in a reticuloendothelial system but also in hepatocytes instead of circulation consequently reducing the availability of this essential element [35].

## 5. Conclusion

From these findings, we can conclude that the use of tacrolimus may lead to the onset of intrahepatic APR-like reaction and causes iron overload in hepatic cells by altering the expression of key proteins involved in iron metabolism. This study established a facet to the concept that despite being essential on the one hand, this drug can also contribute to posttransplant iron deficiency and anemia due to the accumulation of iron in the liver.

### 5.1. Clinical Perspectives

- We investigated the effect of short-term early exposure of tacrolimus on the gene expression of main proteins involved in iron metabolism
- This study established a facet to the concept that despite being essential on one the hand, this drug can also cause damaging effects through the onset of an intrahepatic acute-phase response-like reaction
- These findings are beneficial and can be used as ready reference when prescribing this medicine for therapeutic purpose

## Figures and Tables

**Table 1:** RT-PCR primer list and sequences.

	Forward 5′⟶3′	Reverse 5′⟶3′
*Hepc*	GAA GGC AAG ATG GCA CTA AGC A	TCT CGT CTG TTG CCG GAG ATA G
*HO-1*	CAA CCC CAC CAA GTT CAA ACA G	AAG GCG GTC TTA GCC TCT TCT G
*Fpn1*	TTC CGC ACT TTT CGA GAT GG	TAC AGT CGA AGC CCA GGA CTG T
*Hjv*	ATG CCG TGT CCA AGG AGC TT	TCC ACC TCA GCC TGG TAG AC
*Heph*	CAC ATT TTT CCA GCC ACC TT	TGA CGA ACT TTG CCT GTG AG
*Tf*	GGC ATC AGA CTC CAG CAT CA	GCA GGC CCA TAG GGA TGT T
*TfR1*	ATA CGT TCC CCG TTG TTG AGG	GGC GGA AAC TGA GTA TGG TTG A
Ferritin-H	GCC CTG AAG AAC TTT GCC AAA T	TGC AGG AAG ATT CGT CCA CCT
Ferritin-L	AAC CAC CTG ACC AAC CTC CGT A	TCA GAG TGA GGC GCT CAA AGA G
*TNF-α*	ACA AGG CTG CCC CGA CTA T	CTC CTG GTA TGA AGT GGC AAA TC
*IL-6*	GTC AAC TCC ATC TGC CCT TCA G	GGC AGT GGC TGT CAA CAA CAT
*IFN-α*	AGG TAG GGG TGC AGG AAT CT	GCA CAG GGG CTG TGT TTA TT
*IFN-β*	GCC TTT GCC ATT CAA GTG AT	GTC TCA TTC CAC CCA GTG CT
*IFN-γ*	AGT CTG AAG AAC TAT TTT AAC TCA AGT AGC AT	CTG GCTC TCA AGT ATT TTC GTG TTA C
*β*-Actin	TGT CAC CAA CTG GGA CGA TA	AAC ACA GCC TGG ATG GCT AC

## Data Availability

The data used to support the findings of this study is available from the corresponding author upon request.
